# Effects of high intensity interval training on neuro-cardiovascular dynamic changes and mitochondrial dysfunction induced by high-fat diet in rats

**DOI:** 10.1371/journal.pone.0240060

**Published:** 2020-10-23

**Authors:** Silvio R. Marques Neto, Raquel C. Castiglione, Teresa C. B. da Silva, Lorena da S. Paes, Aiza Pontes, Dahienne F. Oliveira, Emanuelle B. Ferraz, Carla Christina Ade Caldas, José Hamilton M. Nascimento, Eliete Bouskela

**Affiliations:** 1 Laboratory for Clinical and Experimental Research on Vascular Biology (BioVasc), Biomedical Center, State University of Rio de Janeiro, Rio de Janeiro, RJ, Brazil; 2 Physical Activity Sciences Laboratory (LACAF), Physical Activity Sciences Postgraduate Program, Salgado de Oliveira University (UNIVERSO), Niteroi, RJ, Brazil; 3 Exercise Physiology Laboratory, School of Physical Education, Estácio de Sá University, Rio de Janeiro, RJ, Brazil; 4 Health Science School, Universidade do Grande Rio, Duque de Caxias, RJ, Brazil; 5 Cardiac Electrophysiology Laboratory, Carlos Chagas Filho Institute of Biophysics, Federal University of Rio de Janeiro, Rio de Janeiro, RJ, Brazil; Max Delbruck Centrum fur Molekulare Medizin Berlin Buch, GERMANY

## Abstract

**Background and aims:**

Mitochondrial swelling is involved in the pathogenesis of many human diseases associated with oxidative stress including obesity. One of the strategies for prevention of deleterious effects related to obesity and overweight is engaging in regular physical activity, of which high intensity interval training (HIIT) is efficient in promoting biogenesis and improving the function of mitochondria. Therefore, our aims were to investigate the effects of HIIT on metabolic and neuro-cardiovascular dynamic control and mitochondrial swelling induced by high-fat diet (HFD).

**Methods and results:**

Twenty-three male Wistar rats (60 – 80g) were divided into 4 subgroups: control (C), HIIT, HFD and HFD+HIIT. The whole experimentation period lasted for 22 weeks and HIIT sessions were performed 5 days a week during the last 4 weeks. At the end of the experiments, fasting glucose and insulin tolerance tests were performed. Cerebral microcirculation was analyzed using cortical intravital microscopy for capillary diameter and functional density. Cardiac function and ergoespirometric parameters were also investigated. Mitochondrial swelling was evaluated on brain and heart extracts. HFD promoted an increase on body adiposity (p<0.001), fasting glucose levels (p<0.001), insulin resistance index (p<0.05), cardiac hypertrophy index (p<0.05) and diastolic blood pressure (p<0.05), along with worsened cardiac function (p<0.05), reduced functional cerebral capillary density (p<0.05) and its diameter (p<0.01), and heart and brain mitochondrial function (p<0.001). HFD did not affect any ergoespirometric parameter. After 4 weeks of training, HIIT was able to improve cardiac hypertrophy index, diastolic blood pressure, cerebral functional capillary density (p<0.01) and heart and brain mitochondrial swelling (p<0.001).

**Conclusion:**

In animals subjected to HFD, HIIT ameliorated both cerebral mitochondrial swelling and functional capillary density, but it did not improve cardiovascular function suggesting that the cardiovascular dysfunction elicited by HFD was not due to heart mitochondrial swelling.

## Introduction

Obesity is an important public health problem, reaching global epidemic proportions. The main causes of obesity and overweight are an increase in caloric intake and sedentary lifestyle [1]. For these reasons, obesity is associated with increased prevalence of dyslipidemia, insulin resistance (IR), diabetes *mellitus* type 2 (T2DM) and cardiovascular diseases (CVDs) [2].

The deleterious health effects related to obesity are initially based on development of low-grade inflammatory process, which in turn promotes renin-angiotensin-aldosterone activation (RAAS), endothelial dysfunction, insulin resistance and increased activity of the sympathetic nervous system (SNS), leading to early autonomic dysfunction [3].

Neurocardiovascular diseases have as primary pathology neural changes of the autonomic nervous system and its regulated organs, for example heart and blood vessels. Among the cardiovascular risk factors depending on neural cardiovascular control are stress-induced dysfunctions such as obesity [4].

It should be pointed out that the nervous system and skeletal and cardiac muscles are tissues with great oxidative capacity seriously affected by metabolic diseases, being mitochondrial dysfunction strongly correlated with these pathologies. Mitochondrial swelling is involved on the pathogenesis of many human diseases associated with oxidative stress including obesity [5].

The main strategy for prevention of deleterious effects related to obesity and overweight is healthy lifestyle by engaging in regular physical activity [1]. The American College of Sports Medicine Guidelines [6] suggests performing 30 minutes or more of physical exercise of moderate intensity at least 5 days a week or 20 minutes of vigorous intensity physical activity at least 3 days a week to promote neuro and cardioprotector effects.

Regular moderate-intensity physical activity is a well-known strategy to promote positive effects on BMI and fat mass accumulation resulting in clearly benefit for health and mainly for prevention of CVDs. Recent studies have shown that regular practice of high-intensity interval training (HIIT) is capable of promoting significant improvements on maximal aerobic capacity (VO_2max_) and body composition of adult individuals of both sexes, independently of the level of fitness [7]. In addition, recent studies showed that HIIT is efficient in promoting biogenesis and improving the function of mitochondria [8–10].

Although HIIT promotes benefits to health and fitness these effects seem to be more relevant when assciated to a balanced diet but when associated to a high-fat diet, the results are not as effective [11].

Taking all into account, our aims were to investigate the effects of HIIT associated or not to a high-fat diet in a perspective of integrative physiology based on metabolic and neurocardiovascular dynamic control.

## Materials and methods

### Animals

The study followed the Principles of Laboratory Animal Care published by US National Institute of Health (NIH publication, revised in August 2002) and was approved by the State University of Rio de Janeiro Committee for Animal Experimentation (CEUA 060/2012). All invasive procedures were performed under ketamine (90 mg.kg^-1^, i.p.; Venco, PR, Brazil) and xylazine anesthesia (10 mg.kg^-1^, i.p.; Syntec, SP, Brazil).

Recently weaned male Wistar rats (n = 23) were divided into two initial groups: high-fat diet (n = 11) and lean control group fed with standard chow (Nuvilab CR-1, Quimtia S.A, Nuvilab®, Colombo, PR, Brazil) (n = 12). The composition of both diets were previously described by Marques-Neto and co-workers (Table 1) [5]. During the whole experimental period, the animals were housed in a temperature-controlled room (23±2°C) on a 12:12 h dark/light cycle with free access to its respective chow and water.

**Table 1:** Composition of standard chow and high-fat diet.

Ingredients	Standard chow		High-fat diet	
	g kg^-1^	kcal kg^-1^	g kg^-1^	kcal kg^-1^
Cornstarch (Q.S.P)	530	2120	294	1178
Casein	220	880	220	880
Ether Extract	40	360	40	360
Lard	-----	-----	235	2115
Soybean Oil	70	630	70	630
Cellulose	80	-----	80	-----
Mineral mix	35	-----	35	-----
Vitamin mix	10	-----	10	-----
L-Cystine	3	-----	3	-----
Choline	2.5	-----	2.5	-----
Total	1000	3990	1000	5163

After 18 weeks, each initial group was subdivided into two subgroups, generating four experimental subgroups: control group with standard diet and without physical activity intervention (C, n = 6), control group standard diet and HIIT (HIIT, n = 6), high-fat diet group without physical activity intervention (HFD, n = 6), and high-fat diet group with HIIT (HFD+HIIT, n = 5).

### Adaptation to HIIT

Initially, rats ran on a motor-driven treadmill (AVS Projetos, São Carlos, SP, Brazil) during 5 days for 10 min to adapt to treadmill running. During exercise, animals ran at 0° inclination with an initial speed of 6 m/min on the motor-driven treadmill. The speed was increased by 1 m.min^-1^ every 1 min until exhaustion. During the test, maximal oxygen uptake (VO_2max_) was measured while animals ran on the treadmill connected to a metabolic chamber via a capillary tube. The airflow inside the chamber was maintained by an air pump (3,500 ml.min^-1^). The gas collected during the tests was transferred to a gas analyzer (OXYLET SYTEM-Treadmill, Panlab, Havard Apparatus, MA, USA), and oxygen uptake was then calculated (VO_2_ = pO_2_ room air—pO_2_ during test x F m^-1^), where F is the airflow in the chamber (3,500 ml.min^-1^) and m is rat’s body mass in kilograms.

### Ventilatory thresholds and maximal oxygen uptake

Ventilatory threshold (VT) and respiratory compensation (RC) point were automatically determined by searching for breakpoints of carbon dioxide production (VCO_2_). VCO_2_ was modeled by fitting three contiguous linear segments, and first (VT) and second (RC) breakpoints were obtained, as previously described in humans [12, 13]. VO_2max_ was considered as achieved when VO_2_ did not increase with increasing workload and rats achieved exhaustion.

### HIIT protocol

After 18 weeks of standard chow or high-fat diet, both subgroups were exercised 5 days a week for 4 weeks on a motor-driven treadmill (AVS Projetos, São Carlos, SP, Brazil). The training session consisted of a 5-min warm-up followed by 3-min run at 85% of VO_2max_ and by 3-min intervals at 60% of VO_2max_. This sequence was repeated 7 times during the training sessions and lasted about 47 min [14].

### Fasting glucose and insulin tolerance test

Forty-eight hours after the last training session rats were subjected to a 6-hour fasting and glucose and insulin tolerance (ITT) tests were performed [15–17]. Fasting glucose was quantified using the glucose meter (OneTouch^®^ Ultra^®^, Milpitas CA, USA). Five minutes after the first glucose collection, 1.5 IU kg^−1^ of human recombinant insulin (Novolin^®^, Novo Nordisk A/S, Kalundborg, Denmark) was injected i.p. and then a drop of blood from the tail were collected at 0, 5, 10, 15, 20, 25 and 30 min for serum glucose determination. The rate constant for plasma glucose disappearance (k_ITT_) was calculated as previously described [18]. Twenty-four hours after glucose and insulin tolerance (ITT) tests animals were euthanized.

### Cerebral microcirculation

Cerebral microcirculation experiments were performed at the end of the experimental period, as previously described by Marques-Neto and co-workers [5]. Briefly, rats were anesthetized with ketamine (90 mg.kg^-1^, i.p.; Venco, PR, Brazil) and xylazine anesthesia (10 mg.kg^-1^, i.p.; Syntec, SP, Brazil) and placed in a stereotaxic apparatus (AVS Projetos, São Carlos, SP, Brazil). A cranial and relatively large window (4 x 6 mm^2^) was generated through an electric powered drill (Beltec, LB-100, Araraquara, SP, Brazil), the bone removed and dura mater resected for optical access. The window was sealed and a PE-10 catheter inserted into the femoral vein to deliver 0.4 mL volume of 5% fluorescein-dextran (FITC-dextran 150) dissolved in saline to labeled blood serum (2MDa; Sigma Aldrich, St. Louis, Missouri, USA) [19].

Sequentially, cortical intravital microscopy (AxioScopeA1, Carl Zeiss, Oberkochen, Germany) was carried out and images of parietal cerebral microcirculation were acquired using an AxioVision software 4.7 (Carl Zeiss, Oberkochen, Germany). Only perfused capillaries were counted to determine mean functional capillary density, expressed as number of capillaries/mm^2^.

### Arterial blood pressure and ventricular function

After cerebral microcirculation and intravital microscopy protocols, a polyethylene catheter (PE-50) filled with heparinized saline was inserted into the right carotid artery for systolic (SBP) and diastolic (DBP) arterial blood pressure recording. After arterial blood pressure measurements, the catheter was introduced into the left ventricle (LV) for measurement of systolic and diastolic pressures and their first-time derivatives (positive and negative, maximum dP/dt^+^ and minimum dP/dt^-^, respectively).

All arterial and ventricular measurements were registered in real-time and continuously recorded in a four-channel data acquisition system (PowerLab 4/35, ADInstruments, Colorado, CO, USA). After, signals were analyzed off-line using LabChart software (version 7.0, AD Instruments, Colorado Springs, CO, USA). All data were measured for ≥5 min. After these evaluations, the anesthetized rats were decapitated and their tissues (when necessary) weighted and designed to mitochondrial isolation and swelling assay.

### Analysis of the Heart Rate Variability (HRV)

HRV signals processing was done using Matlab-based algorithms, as previously described [12, 20]. Briefly, for spectral analysis of HRV, tachograms were resampled to equal intervals by spline cubic interpolation method at 10 Hz, and the linear trend was removed. Power spectrum was obtained with a fast Fourier transform based method (Welch’s periodogram: 256 points, 50% overlap, and Hamming window). In the time domain, the following indexes were obtained: mean R–R interval (R–R), square root of mean squared differences of successive R–R intervals (RMSSD) and percentage of successive R–R interval differences greater than 5 ms (pNN5). For spectral (frequency domain) analysis of HRV, both low-frequency (LF: 0.2–0.8 Hz) and high-frequency (HF: 0.8–2.5 Hz) bands were determined. The LF/HF ratio was used as an index of sympathovagal balance, as described previously [20, 21].

### Mitochondrial isolation and swelling protocols

Mitochondria from rat brains and hearts were isolated as previously described [22]. Brains and hearts were homogenized in isolation buffer (250 mM Sucrose, 1 mM EGTA, 5 mM HEPES, pH 7.3; all from Merck Millipore, Darmstadt, Germany) containing 1 mg of subtilisin A (Sigma Aldrich, St. Louis, Missouri, USA) per g of tissue and centrifuged at 1,000 g for 10 min at 4°C. The pellet was discarded and the supernatant centrifuged again at 12,000 g for 15 min. Briefly, 1 mg/ml of heart mitochondria were suspended in mPTP buffer (200 mM Sucrose, 10 mM HEPES, 5 mM KH_2_PO_4_, 10 μM EGTA, pH 7.3; all from Merck Millipore, Darmstadt, Germany) supplemented with 10 mM succinate (Merck Millipore, Darmstadt, Germany) and 1.5 mM rotenone (Sigma Aldrich, St. Louis, Missouri, USA). Mitochondrial swelling was triggered by addition of 1 mM of CaCl_2_ to brain and heart and monitored by the relative decrease in percentage of light-scattering at 540 nm in a Spectramax M2 spectrophotometer (Molecular Devices, CA, USA) for 21 minutes.

### Statistical analysis

Sample size was calculated using GPower version 3.1 (Heinrich-Heine-Universität Düsseldorf, Germany). All data are presented as mean ± SEM. Cardiorespiratory parameters (VT, RC and VO2max) were compared before (week 18) and after the 4 weeks of HIIT (week 22) for each experimental group with Students t-test. All other statistical analysis were performed with *Two-way* analysis of variance (ANOVA), with HFD as one factor (F_1_) and HIIT as the second factor (F_2_), followed by Tukey *post hoc* test for multiple comparisons. Mitochondrial swelling from all groups was analyzed together but represented separately for a better visualization of the results. Significance level was established at *p<*0.05.

## Results

### Biometric results

Table 2 illustrates biometric results of experimental subgroups. After 18 weeks of intervention, no differences were observed on body weight between them.

**Table 2:** Biometric parameters after diet/high intensity interval training. Values as Mean±SEM.

Variables	Groups				Factors		
	C	HFD	HIIT	HFD+HIIT	F_1_	F_2_	I
BW (g)	420.4±14.1	457.0±18.3	451.5±5.9	461.2±25.1	ns	ns	ns
HW (g)	1.15±0.03	1.54±0.05***	1.37±0.07*	1.46±0.06**	p<0.001	ns	p<0.05
HW/BW (g/kg)	2.74±0.12	3.39±0.18*	3.00±0.13	3.30±0.20	p<0.01	ns	ns
EF (g)	5.52±0.94	11.21±1.50**	4.67±0.30^**###**^	11.56±0.96^**$ $**^	p<0.0001	ns	ns
RF (g)	5.58±1.85	15.93±1.62***	4.04±0.28^**####**^	16.77±1.80^**$ $ $**^	p<0.0001	ns	ns
VF (g)	3.82±0.17	11.69±1.23***	4.05±0.34^**###**^	12.93±1.72^**$ $ $ $**^	p<0.0001	ns	ns

BW (Body weight), HW (Heart weight), EF (Epididymal fat), RF (Retroperitoneal fat) and VF (Visceral fat).

*p < 0.05 vs. C

**p < 0.01 vs. C

***p <0.001 vs. C

^###^*p* <0.001 vs. HFD

^####^*p* <0.0001 vs. HFD

^**$ $**^*p* <0.01 vs. C and HIIT

^**$ $ $**^*p* <0.001 vs. C and HIIT

^**$ $ $ $**^*p* <0.0001 vs. C and HIIT. ns, no significant difference for the probability based on the Tukey’s post hoc analysis. F1: HFD factor; F2: HIIT factor; and I: interaction between F1 and F2 for the probability based on a two-way analysis of variance.

To investigate whether heart weight and cardiac hypertrophy index [obtained from body weight (BW) and heart weight (HW) relationship] could be affected by HIIT, both of them were evaluated in the experimental subgroups. Heart weight of HFD, HIIT and HFD+HIIT subgroups was significantly higher than in C one but cardiac hypertrophy index was higher only in HFD when compared to C.

To further investigate whether HIIT could modulate regional fat accumulation, fat weight depots were determined in all experimental subgroups. After 22 weeks, HFD and HFD+HIIT animals presented similar epididymal, retroperitoneal and visceral fat depots but larger than in C and HIIT rats.

### Fasting glucose and insulin tolerance test

Table 3 shows the results of blood glucose and insulin tolerance tests. After 22 weeks of high-fat diet, increased fasting glucose levels and higher insulin resistance index (KITT and AUC) during ITT were observed on HFD and HFD+HIIT subgroups compared with C and HIIT ones.

**Table 3:** Fasting glucose and insulin tolerance test (ITT) parameters after high-fat diet (HFD) and high intensity interval training (HIIT). Values as Mean±SEM.

Variables	Groups				Factors		
	C	HFD	HIIT	HFD+HIIT	F_1_	F_2_	I
***Glucose (mg/dL)***							
Fasting	86.5±3.39	115.0±2.89***	93.5±3.43^**##**^	115.5±5.30^**$ $**^	p<0.0001	ns	ns
***ITT parameters***							
K_ITT_ (%/min)	4.40±0.75	2.09±0.12*	4.70±0.65^**##**^	2.03±0.17^**$ $**^	p<0.0001	ns	ns
AUC	2622±219	3585±174**	2491±101^**###**^	3351±83^**$ $**^	p<0.0001	ns	ns

*p < 0.05 vs. C

***p <0.001 vs. C

^##^*p* <0.01 vs. HFD

^**$ $**^*p* <0.01 vs. C and HIIT

^**$ $**^*p* <0.01 vs. C and HIIT

ns, no significant difference for the probability based on the Tukey’s post hoc analysis. F1: HFD factor; F2: HIIT factor; and I: interaction between F1 and F2 for the probability based on a two-way analysis of variance.

### Cardiorespiratory parameters during maximal exercise testing

**Fig 1:**
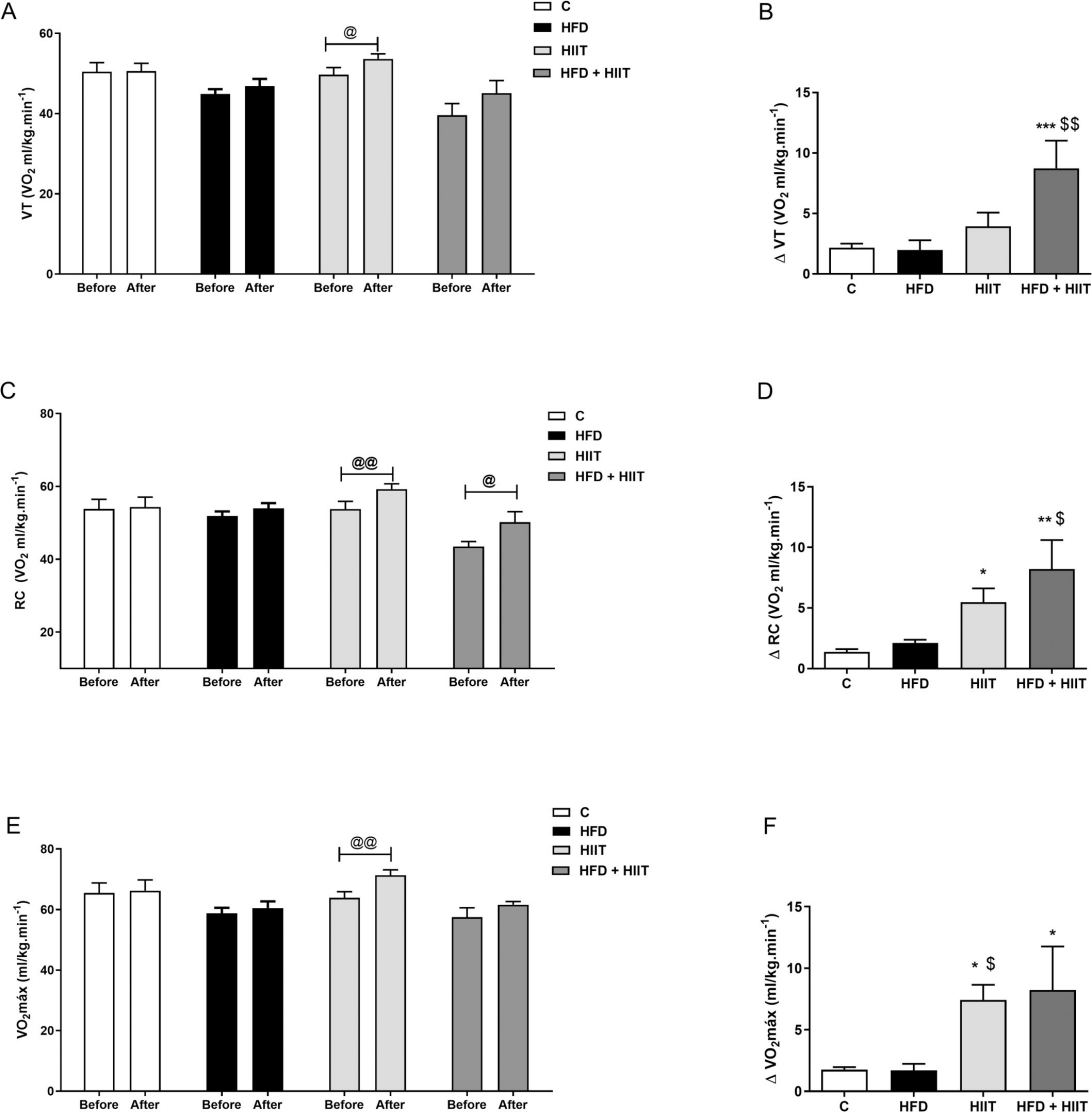
Changes on cardiorespiratory parameters during maximal exercise before and after 4 weeks of HIIT training. In (a): ventilatory thresholds (VT) before and after HIIT, (b) variation of VT, (c) respiratory compensation (RC) before and after HIIT, (d): variation of RC, (e) VO_2max_ before and after HIIT and (f): variation of VO_2max_. Data are shown as mean ± SEM; n = 5–6. @p<0.05 vs. before; @@p<0.01 vs. before; *p<0.05 vs. C; **p<0.01 vs. C; ***p<0.001 vs. C; ^$^p<0.05 vs. HFD; ^$ $^p<0.01 vs. HFD.

Cardiorespiratory parameters were compared before (week 18) and after the 4 weeks of HIIT (week 22) for each group (Fig 1A, 1C and 1E). We can observe an increase in VT, RC and VO_2max_ in HIIT group, showing that exercise is able to improve physical performance. In HFD+HIIT group, only RC levels were increased, indicating that HIIT promotes an increase in fatigue resistance in animals fed a high-fat diet, without improvement of LV and VO_2max._

After 4 weeks of training, HFD+HIIT animals had an improvement on VT (Fig 1B; F_1:_ p<0.05 and F_2:_ p<0.001), RC (Fig 1D; F_2:_ p<0.001) and VO_2max_ (Fig 1F; F_2:_ p<0.001) compared to both C and HFD subgroups. Interestingly, only VO_2max_ was increased in HIIT compared to C group.

Our results also suggest that in our animal model high-fat diet does not change the cardiopulmonary capacity improvement after exercise in relation to lean exercised animals.

### Haemodynamics and left ventricular (LV) parameters

**Fig 2:**
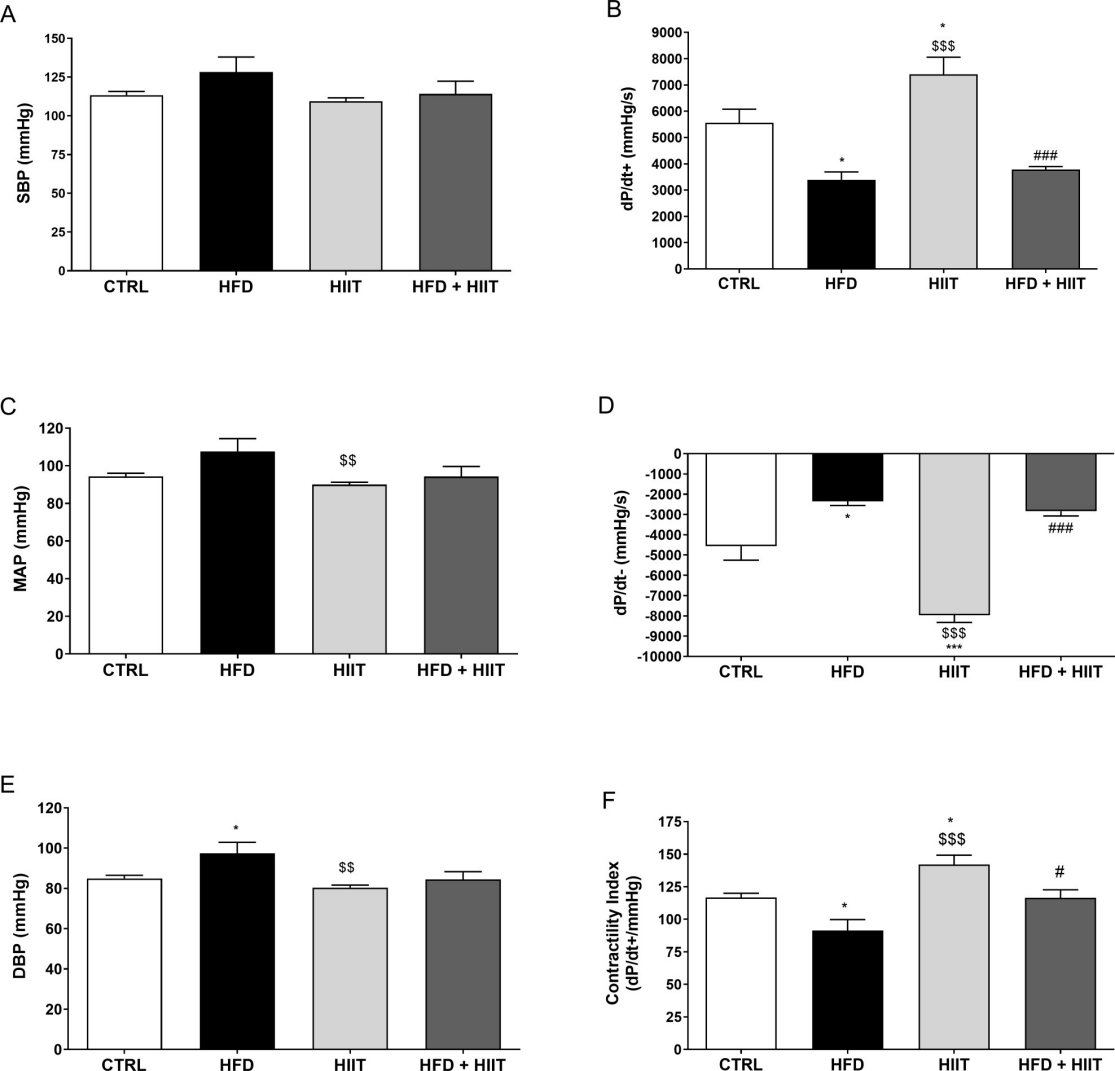
Haemodynamic and LV parameters after HFD and HIIT. In (a): systolic blood pressure; (b): dP/dt+; (c): mean arterial pressure; (d): dP/dt-; (e): diastolic blood pressure and (f): contractility index values. Data are shown as mean ± SEM; n = 5–6. *p<0.05 vs. C; ***p<0.001 vs. C; ^$ $^p<0.01 vs. HFD; ^$ $ $^p<0.001 vs. HFD; ^#^p<0.05 vs. HIIT; ^###^p<0.001 vs. HIIT.

The results of haemodynamic and LV function are summarized on Fig 2. At the end of the experimental period, the HFD subgroup showed higher diastolic blood pressure (F_1_: p<0.05 and F_2_: p<0.05), reduced values of contractility (dP/dt+; F_1_: p<0.0001 and F_2_: p<0.05), contractility index (F_1_: p<0.001 and F_2_: p<0.001) and relaxation (dP/dt-; F_1_: p<0.0001, F_2_: p<0.01 and I: p<0.01) compared to C and control HIIT subgroups.

Although HFD animals showed a 15% increase in SBP and 14% increase in MBP compared to C groups, no statistical differences were observed in these parameters.

After 4 weeks of training, the control HIIT subgroup presented higher value of contractility and relaxation index than the C one. The animals trained and fed with high-fat diet (HFD+HIIT) showed reduced values of contractility and relaxation indexes compared to HIIT and similar values to C and HFD.

### Analysis of the heart rate variability

**Fig 3:**
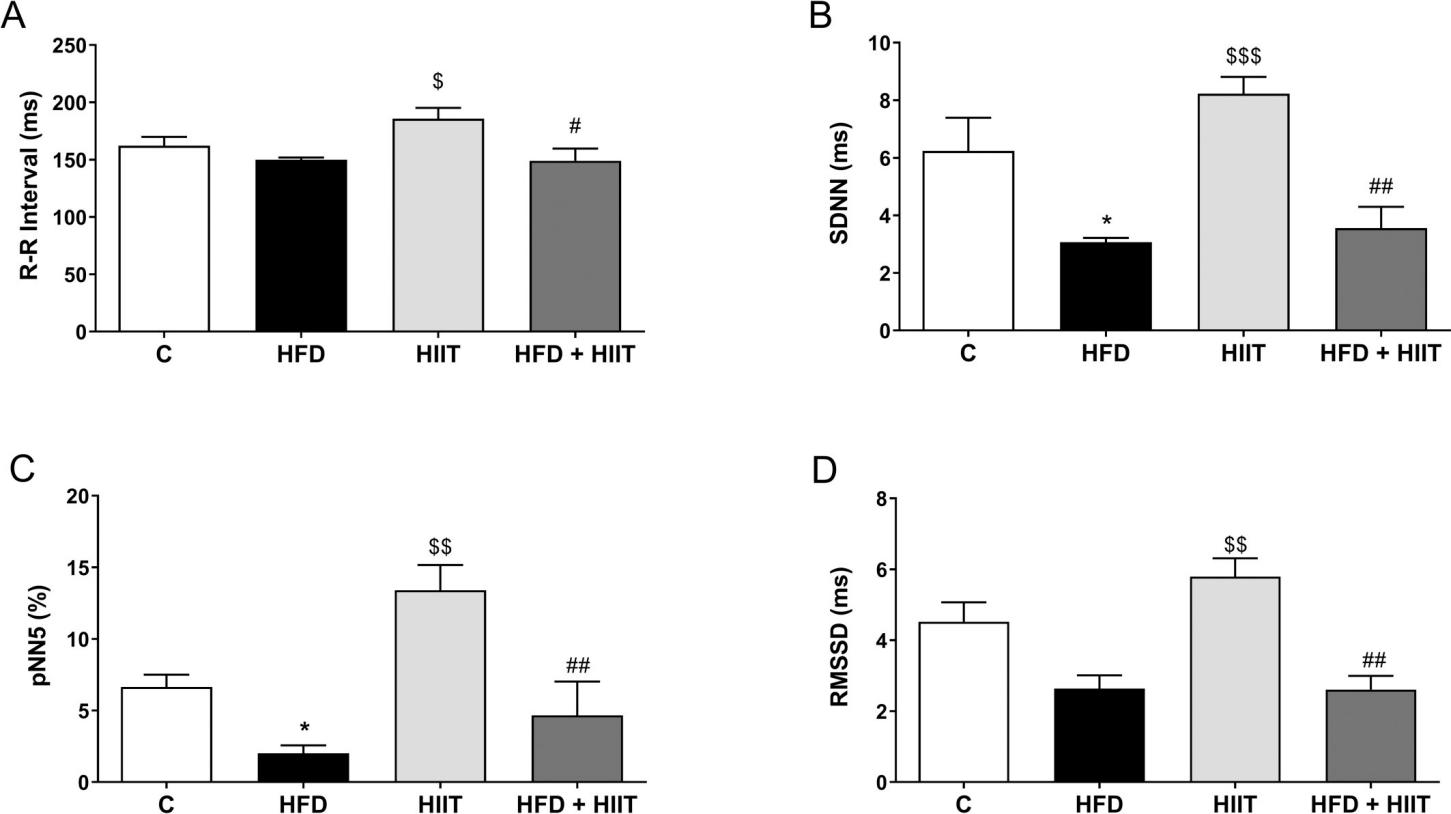
Time-domain analysis of HRV after HFD and HIIT. In (a): R-R interval; (b): SDNN; (c): pNN5 and (d): RMSSD. Data are shown as mean ± SEM; n = 5–6. *p<0.05 vs. C; ^$^p<0.05 vs. HFD; ^$ $^p<0.01 vs. HFD; ^$ $ $^p<0.001 vs. HFD; ^#^p<0.05 vs. HIIT; ^##^p<0.01 vs. HIIT.

Time-domain indexes of HRV (SDNN, F_1_: p<0.001; and pNN5%, F_1_: p<0.01 and F_2_: p<0.05) were reduced in HFD group compared with C. HFD + HIIT had reduced values compared to HIIT, showing that high-fat diet is a limiting factor for HIIT benefits (Fig 3).

Regarding R-R intervals (F_1_: p<0.05) and RMSSD (F_1_: p<0.001) values HIIT has higher values than HFD and lower values than HFD + HIIT.

**Fig 4:**
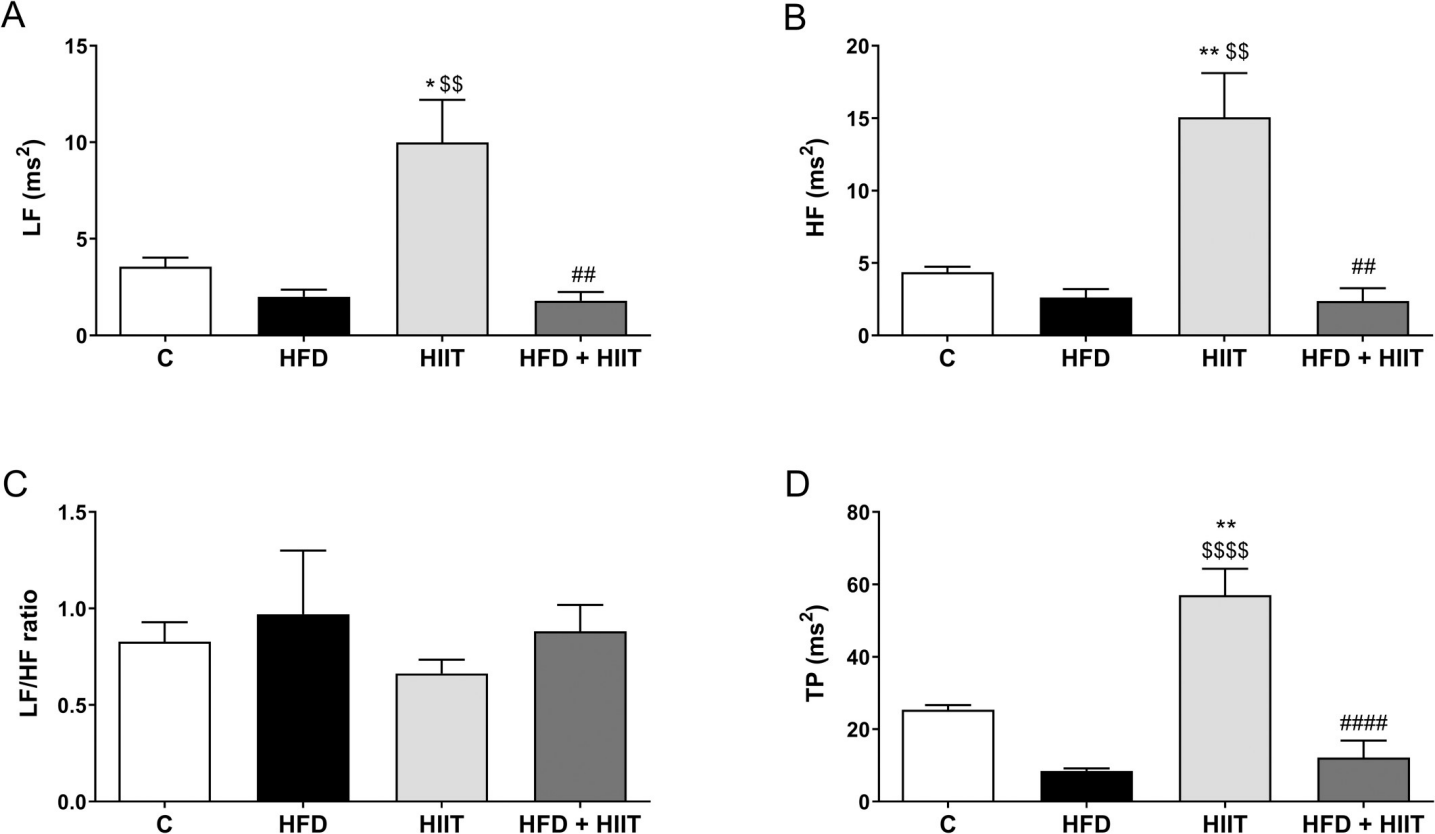
Power spectra indexes of HRV after HFD and HIIT. In (a): LF; (b): HF; (c): LF/HF and (d): TP. Data are shown as mean ± SEM; n = 5–6. *p<0.05 vs. C; **p<0.01 vs. C; ^$ $^p<0.01 vs. HFD; ^$ $ $ $^p<0.0001 vs. HFD; ^##^p<0.01 vs. HIIT; ^####^p<0.0001 vs. HIIT.

Additionally, power spectra indexes of HRV (LF, F_1_: p<0.01 and I: p<0.05; HF, F_1_: p<0.01, F_2_: p<0.05 and I: p<0.05; and TP, F_1_: p<0.0001, F_2_: p<0.01 and I: p<0.05) were significantly higher in HIIT compared to C and HFD groups. Also, HIIT protocol executed in HFD animals did not improve these parameters when compared to HFD group (Fig 4).

Taken together, these results show that HFD group has an autonomic dysfunction compared to C group and that HIIT was not able to improve these parameters in animals fed a high-fat diet.

### Cerebral microcirculation

**Fig 5:**
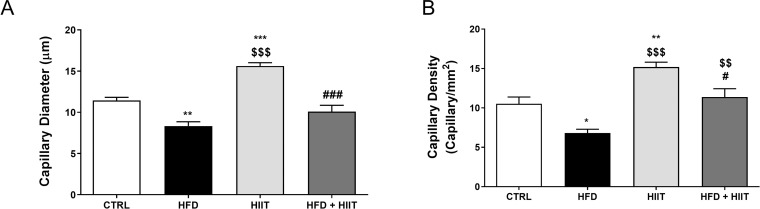
Microcirculation analysis of brain capillaries after HFD and HIIT. In (a): Capillary diameter and in (b): functional capillary density. Data are shown as mean ± SEM; n = 5–6. *p<0.05 vs. C **p<0.01 vs. C; ***p<0.001 vs. C; ^$^p<0.05 vs. HFD; ^$ $ $ $^p<0.0001 vs. HFD; ^##^p<0.01 vs. HIIT; ^####^p<0.0001.

As shown on Fig 5, capillary diameter (F_1_: p<0.0001, F_2_: p<0.0001 and I: p<0.05) and capillary functional density (F_1_: p<0.001 and F_2_: p<0.0001) were reduced on HFD compared to C rats. HIIT training in animals fed standard chow was able to increase both parameters, however in rats fed high-fat diet, the same training only improved functional capillary density, reaching control levels.

### Mitochondrial swelling

**Fig 6:**
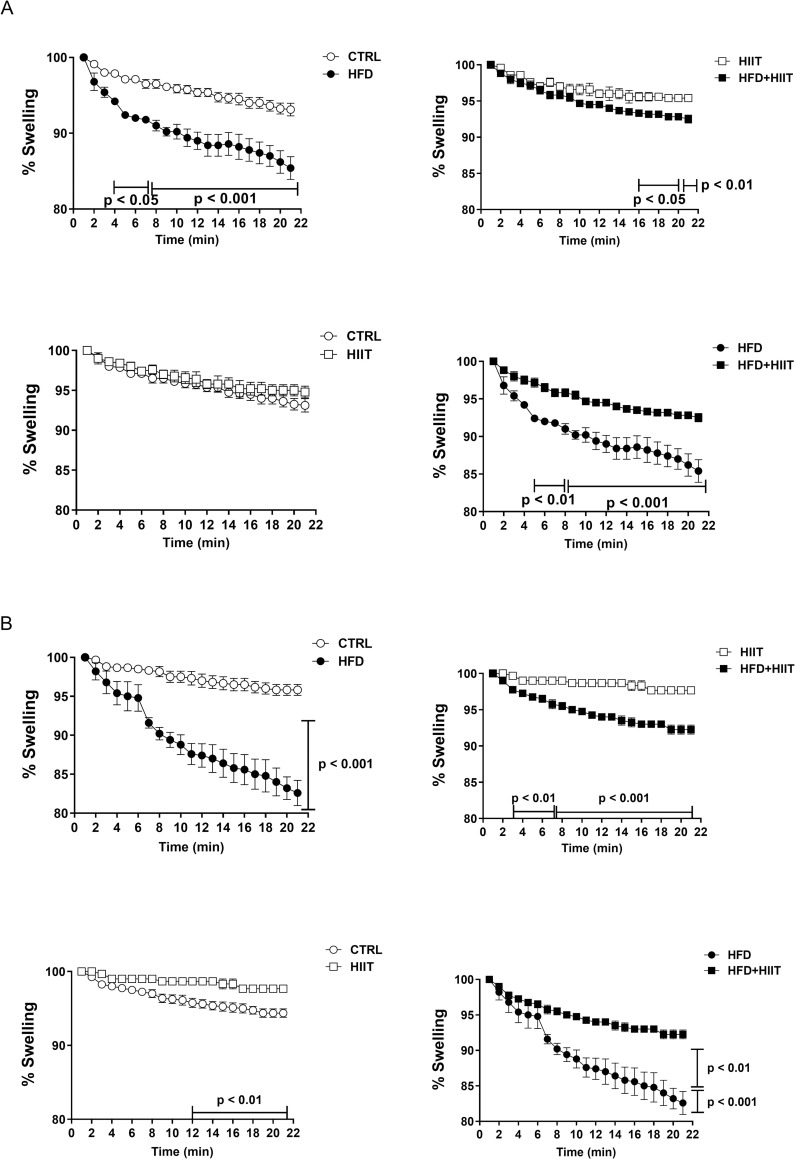
Experimental swelling protocol induced by Ca_2_^+^. mPTP resistance in brain (a) and heart (b) tissues. Analyses were performed in rats exercised with HIIT training for 4 weeks after high-fat diet for 18 weeks. Data are shown as mean ± SEM; n = 5.

To determine mitochondrial permeability transition pores (mPTP) function from cerebral cortex and LV, mitochondrial swelling was measured in response to Ca_2_^+^ (Fig 6).

Both cortical (time: p<0.0001, F_1_: p<0.0001 and time vs. F_1_: p<0.0001) and LV (time: p<0.0001, F_1_: p<0.0001 and time vs. F_1_: p<0.0001) mitochondria from HFD rats showed higher swelling than the C group. Notably, HFD+HIIT mitochondria were more resistant to swelling and permeability transition induced by Ca^2+^ than HFD mitochondria in both cerebral cortex (time: p<0.0001, F_2_: p<0.001 and time vs. F_2_: p<0.0001) and LV (time: p<0.0001, F_2_: p<0.001 and time vs. F_2_: p<0.0001). On the other hand, LV mitochondrial swelling of HIIT rats were increased compared to C rats (time: p<0.0001, F_2_: p<0.05 and time vs. F_2_: p<0.0001).

It is interesting to notice that although mitochondrial swelling of HFD+HIIT was lower than in HFD rats, it was still increased compared to HIIT group in LV (time: p<0.0001, F_1_: p<0.001 and time vs. F1: p<0.0001) and cerebral cortex (time: p<0.0001, F_1_: p<0.05 and time vs. F1: p<0.0001).

## Discussion

Excessive intake of fat in diets negatively affects brain and cardiovascular functions, and the metabolic disorders related to cerebral and cardiovascular systems are strongly correlated with mitochondrial dysfunction, responsible for the development of ischemic diseases in these tissues [6, 23, 24].

Unlike some studies in the literature [25–28] the present study did not show significant differences in body weight after 22 weeks of a high-fat diet. This finding may be explained by the fact that animals belonging to this group have ingested a lower absolute amount of the chow compared to the group with a normal diet and, therefore, both groups have ingested a similar amount of calories (data not shown).

On the other hand, it is noted that fat deposits increased significantly in groups submitted to a high-fat diet. In this sense, the present study suggests that even though there were no changes in body weight between groups, the high-fat diet was responsible for reducing lean body mass and developing insulin resistance, regardless of physical training.

Although major findings in the literature show that HIIT is able to modulate these parameters [29–31], we did not observe any improvement in our animals after 4 weeks of HIIT training. Our results are in accordance with Keating and co-workers [30], that suggest that in previously inactive, overweight adults, 12 weeks of HIIT is able to improve fitness but it is not a time-effective mean of increasing fat loss or improving fat distribution.

Additionally, it is known that the high-fat diet and obesity are characterized by an important cardiac remodeling [32]. Leopoldo et al. [33] showed that the high-fat diet was responsible for increasing left ventricular mass, reducing systolic function and collagen deposition in the heart, a fact associated with its pathological remodeling. In our study, although the hypertrophy index only showed differences in the HFD (sedentary) group, the absolute heart weight of the HIIT, HFD and HFD + HIIT groups was greater than the control group.

Besides cardiac remodeling, obesity has several consequences on hemodynamic parameters, related to the increase in blood pressure and myocardial dysfunction. In this sense, we investigated whether changes in heart weight were in fact associated with myocardial and hemodynamic dysfunction, and we were able to show that the high-fat diet, associated or not with physical training, was able to reduce contractility and relaxation indexes of the heart, which are associated with reduced myocardial function [32]. These effects can be explained by the increase in left ventricular mass, relative wall thickness and reduction in the internal diameter of the cardiac chamber and wall shortening, as previously reported [32].

Although our results suggest that HIIT is able to reverse cardiac hypertrophy after long-term feeding with high-fat diet, HIIT could only improve haemodynamics and left ventricular function in rats fed with normal chow. The only exception is the reversion of an increase in DBP on HFD rats after training. It is already known that DBP is directly related to peripheral vascular resistance [34] and microvascular dysfunction [35]. Systolic blood pressure is related to heart rate (R-R interval) [36, 37]. Since R-R interval was not modulated by HFD it is expected that SBP does not change with diet the same way. These same patterns were observed in other therapies, such as incretin-based ones [5].

Once metabolic changes occur, as well as changes in the heart structure and myocardial function, the autonomic nervous system needs to act as a compensatory mechanism (feedback) to try to keep the physiological parameters within the normal range [38, 39]. Since these parameters are affected by obesity, there is a development of autonomic dysfunction, an important factor in increasing the risk of sudden death in these subjects [40]. In this sense, by analyzing time-domain parameters of HRV, we can see that the high-fat diet was responsible for the development of autonomic dysfunction independent of physical training (HIIT). On the other hand, as reported previously, HIIT was able to promote benefits in autonomic function after 4 weeks of physical training compared to the control group [41]. These results explain the functional changes observed in the contractility and relaxation indexes of the heart, since autonomic dysfunction is one of the mechanisms associated with remodeling and myocardial dysfunction [42].

In addition to autonomic dysfunction having an influence on myocardial function, increased sympathetic activity also promotes microvascular damage to the brain and heart, which can lead to an increased risk of myocardial infarction and stroke [42]. Microvascular dysfunction has a deleterious impact on different tissues and it is dependent of cellular energetic metabolism homeostasis [43, 44]. It has been suggested that altered cellular metabolism of high-fat diet individuals could allow changes in both cerebrovascular resistance and blood flow [45].

Our findings show that the high-fat diet, not only compromised myocardial function, but also promoted significant deleterious changes in the cerebral microvascular structure, by reducing both diameter and capillary density. HIIT training is able to stimulate brain-derived neurotrophic factor (BDNF) in brain, a protein that plays a key role to maintain or improve several brain functions [46] and to improve oxygen utilization during cortical activation in older individuals [47].

Although HIIT increased these parameters in animals fed a control diet, the diet-associated training group (HFD + HIIT) maintained mean values similar to those of the C group, showing a partial response of these effects compared to HIIT group.

HIIT is able to improve endothelial function, probably because the recovery period between short bouts of HIIT could significantly enhance antioxidant status, indicating decreased oxidative stress and increased NO bioavailability [48].

The fact that neurocardiovascular dynamics is compromised with a high-fat diet may be explained by recent studies that show that autonomic dysfunction, the main factor associated with myocardial and microvascular dysfunction, may be the result of a set of mitochondrial disorders in tissues [49]. These disorders result in reduced ability to consume oxygen, as well as an increase in reactive oxygen species that are responsible by increased mitochondrial calcium overload [42, 49].

During stress, mitochondrial Ca^2+^ overload triggers the additional increase in oxidative stress and the subsequent opening of the inner membrane permeability transition pore (mPTP), together with osmotic swelling and loss of ATP synthesis, leads to cell death of neurons and cardiac myocytes [42, 49]. The results of the present study show that in the face of an acute stress stimulus, like Ca^2+^ overload during mitochondrial swelling assay, the heart and brain mitochondria in the high-fat diet groups showed greater swelling due to the reduction of mitochondrial function.

During high intensity training, the activation of PGC-1α stimulates the expression of nuclear genes encoding mitochondrial proteins that regulate skeletal muscle mitochondrial size, number, composition (protein-to-lipid ratio) content and function [50].

Even with short exercise duration, HIIT enhances mitochondrial biogenesis and also angiogenesis. The induction of HIF1 and VEGF after HIIT may be important to create greater arterio-venous oxygen differences and influence VO_2max_ [51]. The maximal oxygen uptake (VO_2max_) and whole-body aerobic capacity are limited not only by cardiorespiratory factors but also by mitochondrial content [50].

Among many modalities of physical activities, HIIT significantly increases approximately two times cardiorespiratory fitness in comparison to moderated physical activity programs in patients with chronic diseases induced by overweight and obesity [52]. We have shown that indeed HIIT is able to improve ventilatory thresholds and VO_2max_ in animals fed standard chow but not high-fat diet. However, we can observe that differences between VT, RC and VO_2max_ HFD+HIIT animals before training are no longer observed after 4 weeks of HIIT.

In terms of limitations of our study, it is important to consider that we did not evaluate mitochondrial O_2_ consumption, brain function, cerebral microcirculatory blood flow and cerebral vascular inflammation (leukocyte adhesion). Intravital microscopy is a well described technique to observe vessel diameter, but it is limited to vessels on or close to the pial surface, limiting the extent of capillaries analyzed. Future studies should be performed to evaluate mitochondrial O2 consumption, brain function through cognitive tests, cerebral blood flow and inflammation.

## Conclusion

Our results show that high-fat diet reduces cardiovascular function, cerebral capillary density and organic mitochondrial dysfunction. HIIT training on animals subjected to HFD was able to improve both cerebral mitochondrial swelling and functional capillary density, but it was unable to improve cardiovascular function despite improvement on mitochondrial swelling. Therefore, our results suggest that, at least in part, the cardiovascular dysfunction elicited by high-fat diet was not due to heart mitochondrial swelling.
